# Long-Term Efficacy and Tolerability of Abdominal Once-Yearly Histrelin Acetate Subcutaneous Implants in Patients with Advanced Prostate Cancer

**DOI:** 10.1155/2014/490315

**Published:** 2014-12-04

**Authors:** Sean Woolen, Cameron Holzmeyer, Emily Nesbitt, Paul F. Siami

**Affiliations:** ^1^Research Institute of Deaconess Clinic, 421 Chestnut Street, Evansville, IN 47713, USA; ^2^Department of Urology, Deaconess Clinic, 421 Chestnut Street, Evansville, IN 47713, USA

## Abstract

*Objectives.* Long-term assessment of the efficacy and tolerability of subcutaneous abdominal histrelin acetate implants that have been inserted for more than two years. *Materials and Methods.* Retrospective data collected over a six-year period at a single center from charts of 113 patients who received the subcutaneous abdominal histrelin acetate implant. *Results.* Following insertion of the first implant, 92.1% and 91.8% of patients had a serum testosterone level of ≤30 ng/dL at 24 and 48 weeks, respectively. Serum testosterone levels remained at <30 ng/dL for 96% of patients at two years and for 100% of patients at 3, 4, and 5 years. The testosterone levels remained significantly less than baseline (*P* < 0.05). Six patients (5.3%) had androgen-independent progression when followed up on the long term, increasing the mean serum PSA at 3, 4, and 5 years to 35.0 *µ*g/L (*n* = 22), 30.7 *µ*g/L (*n* = 13), and 132.9 *µ*g/L (*n* = 8), respectively. The mean serum PSA was significantly greater than baseline during these years (*P* < 0.05). Eight patients (7.1%) experienced minor, but not serious, adverse events from the histrelin acetate. *Conclusion.* Subcutaneous abdominal histrelin acetate implants are an effective long-term and well-tolerated administration method for treating patients with advanced prostate cancer.

## 1. Introduction

Prostate cancer is a leading cancer among men in the United States (US). The American Cancer Society estimates that, in 2014, there will be over 233,000 new cases of prostate cancer (representing 27% of new cancer cases in men in the US) and over 29,480 deaths caused by the disease (representing 10% of cancer-related deaths) [1]. However, despite the high prevalence and death rates associated with prostate cancer, survival statistics are favorable for patients; the 5-year survival rate of prostate cancer has reached 99.8%, and the cause of death in the majority (87.4%) of patients is due to other disease etiologies [2]. These favorable survival statistics can be attributed to the slow progression of prostate adenocarcinoma as well as its therapeutic management. Currently, disease management options available for low-stage prostate cancer include active surveillance, radical prostatectomy, radiotherapy, and cryotherapy [3].

The management of metastatic and high-risk locally advanced prostate cancer often includes adjuvant therapy with androgen deprivation therapy (ADT) [4]. Testosterone stimulates prostate cancer growth and decreasing androgen hormone levels may slow disease progression [5]. A serum testosterone level <35–50 ng/dL is considered adequate for patients with prostate cancer [6]. The initiation of ADT should be decided individually for each patient based on risks and benefits of treatment [7]. ADT may be achieved surgically (bilateral orchiectomy) or by medical means, including the administration of luteinizing hormone-releasing hormone (LHRH) antagonist or agonist [5, 6, 8].

LHRH agonists are the mainstay therapy for patients with advanced prostate cancer [9]. Following the initial dose, the LHRH agonist stimulates Leydig cells within the interstitium of the gonads to cause a transient increase in testosterone level referred to as the flare phenomenon [5, 10]. Because of the flare phenomenon, LHRH agonist is typically started with an antiandrogen medication, which is subsequently stopped approximately 1 to 3 weeks after commencing therapy. After 1 to 3 weeks, LHRH receptors become desensitized and castration level of testosterone is achieved [5, 10]. LHRH agonists are available in the US as leuprolide, goserelin acetate, triptorelin acetate, and histrelin acetate.

The LHRH agonist histrelin acetate is the only available sustained-release hydrogel implant for once-yearly administration [11]. The once-yearly implant allows patients to receive fewer cycles of implanting and replanting to maintain continuous ADT therapy. The histrelin acetate implant is 3.5 cm long, is 3 mm in diameter, and is administered subcutaneously into the inner aspect of the upper arm or abdomen [12, 13]. The inner aspect of the upper arm can clinically be difficult for the surgeon as it is a mobile insertion site and difficult for elderly patients limited by strength/arthritis to maintain the necessary abducted and extended arm position. Thus, the arm compared to the abdomen site is more difficult for surgeon and is less tolerated by patients.

The long-term efficacy and tolerability of the once-yearly histrelin implant via arm insertion have previously been reported, but the abdomen insertion site has not yet been followed up on the long term to our knowledge. In the pivotal arm implant study, which consisted of an open-label, single-arm study in 138 patients with advanced adenocarcinoma, >99% of patients were observed to have reduced testosterone (<50 ng/dL) during a 52-week period [13]. In an extension of this clinical trial, testosterone levels were maintained at <50 ng/dL for up to 234 weeks in patients who received once-yearly histrelin acetate implants [14]. In a single center retrospective chart review, two-year efficacy and tolerability were demonstrated in 64 patients receiving abdominal histrelin acetate implants; testosterone levels were maintained at <50 ng/dL for 96 weeks in these patients [12]. Thus, long-term efficacy in the arm implantation site and two-year efficacy for the abdominal implant have been observed.

The long-term efficacy of histrelin acetate implants in the abdomen has not been formally evaluated. This study is an extension of the original retrospective chart review of the abdominal implant by Siami and colleagues [12]; eligible patients from the original retrospective chart review as well as patients receiving implants since the last retrospective review were evaluated. The primary objective of this extension study is to assess the long-term efficacy and tolerability of abdominal histrelin implants that have been placed for more than two years. This study is important clinically because it will allow evidence guided abdominal insertion of the histrelin acetate implant for five years, allowing physicians and patients to utilize benefits of the abdominal insertion site.

## 2. Materials and Methods

### 2.1. Subjects and Treatment

Data were collected retrospectively, at a single center during June 2007 to August 2013, from charts of all patients who received the histrelin acetate implant abdominally. Out of 113 patients in this study, 64 were followed up from the original chart review [12] and 49 new patients were added to the study. Patients included in the analysis were aged ≥45 years, were diagnosed with prostate cancer, and were deemed appropriate candidates for ADT. Patients receiving a shorter duration implant with leuprolide acetate or goserelin acetate for less than a year were included in the study. Patients who received prior LHRH antagonist were excluded from the study.

Patients were administered histrelin acetate using a sterile, diffusion-controlled reservoir drug delivery system (VANTAS; Endo Pharmaceuticals, Chadds Ford, PA, USA). The implant contains a histrelin acetate (50 mg) drug core inside a nonbiodegradable cylindrical hydrogel reservoir and releases the drug at ~50 *μ*g/day [11]. During an aseptic office-based surgical procedure, the histrelin acetate implant was inserted subcutaneously into the abdominal area, approximately two fingerbreadths below the costal margins in the midaxillary (nipple) line using the insertion device supplied with the implant.

### 2.2. Ethics

The study was reviewed and found to be compliant with the Deaconess Health System Research Oversight and Privacy Committee policy. Under the NIH HIPAA Privacy Rule, investigators of the study signed a Limited Data Set Use Agreement in order to perform a retrospective review of patients receiving an abdominal histrelin acetate implant. The investigators of this study abide by good clinical practice and regulations in the current revision of the Declaration of Helsinki.

### 2.3. Statistics

Qualitative data from patient characteristics were presented as the number of patients and the percentage of the total population. Quantitative data were summarized using descriptive statistics including mean and standard errors of the mean. Continuous data with testosterone and PSA were analyzed with a standard* t*-test in the statistical software SAS.

## 3. Results

### 3.1. Patient Characteristics

Out of 64 patients from the original chart review, 39 received a second implant, 28 received a third implant, 17 received a fourth implant, and 10 received a fifth implant. A total of 25 patients died after five years, 24 had explantation, five were lost to follow-up, and one stopped treatment due to financial reasons. Out of the 49 new patients, 30 received a second implant, 11 had explantation, seven were lost to follow-up, and one died. Of the individuals who had explantation, the majority of patients decided to switch to intermittent therapy after consulting with a urologist regarding similar efficacy of intermittent and continuous therapy; some had explantation due to decreased libido, and minimal explanted due to experiencing hot flashes/not feeling well. Patient deaths were judged to be unrelated to histrelin acetate implant use.

### 3.2. Demographics

Baseline characteristics of patients according to the chart review were recorded as age, ethnicity, and prior medications. The mean (± standard error of the mean (SEM)) age was 74.91 (±0.98) years. Out of the 100 patients for whom race was recorded, 90 (90.9%) were Caucasians, eight (8.1%) were African Americans, and one (1.0%) was Hispanic. Almost half of the patients (52/114, 44.9%) had been treated with LHRH agonist therapy prior to administration of the histrelin acetate implant; the most common LHRH agonist was leuprolide acetate. Among patients with available serum prostate-specific antigen (PSA) levels prior to the administration of the histrelin acetate implant (97/113), the mean PSA level was 12.1 *μ*g/L, and 55.7% (54/97) of patients had PSA levels ≥5 *μ*g/L. Among patients with available serum testosterone levels prior to the administration of the histrelin acetate implant (33/113), the mean serum testosterone level was 106.6 ng/dL and 48.5% (16/33) of patients had testosterone levels ≤30 ng/dL. Patients in the chart review population who had received prior short acting LHRH agonist therapy received the histrelin acetate implant during the prescribed end period of their prior LHRH agonist administration, and the length of time ranged from 3 months to 1 year of treatment; continued LHRH agonist therapy was indicated for all of these patients. The demographic information is summarized in Table 1.

### 3.3. Efficacy

**Figure 1:**
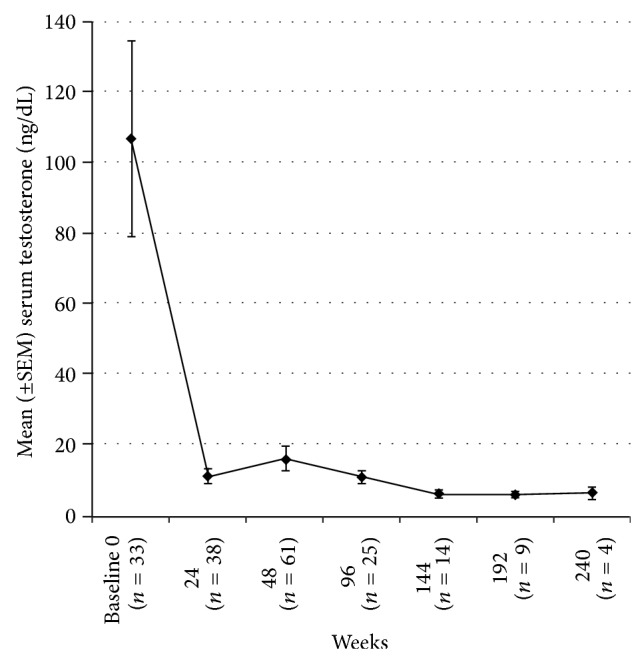
Mean serum testosterone levels (ng/dL) in patients after receiving once-yearly histrelin acetate implants for 240 weeks. The error bars represent the standard error of the mean (SEM).

**Figure 2:**
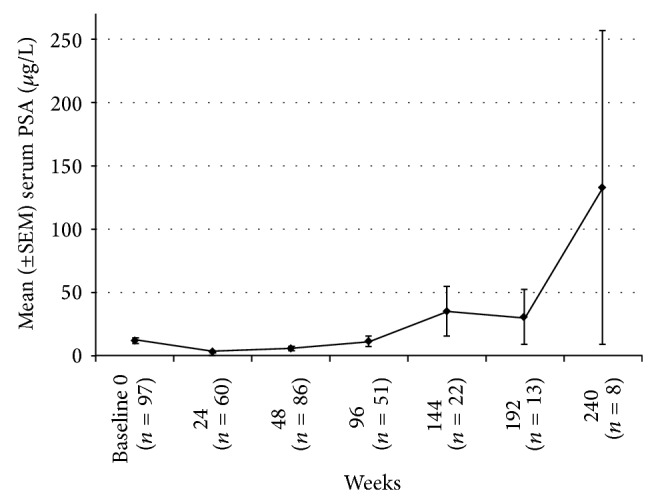
Mean serum prostate-specific antigen (PSA,  *μ*g/L) levels in patients after receiving once-yearly histrelin acetate implants for 240 weeks. The error bars represent the standard error of the mean (SEM).

Following abdominal insertion of the initial implant, the mean serum testosterone level was 11.1 ng/dL at 24 weeks (*n* = 38) and 16.0 ng/dL at 48 weeks (*n* = 61, Figure 1). The percentage of patients with a serum testosterone level ≤30 ng/dL was 92.1% (35/38 patients) at 24 weeks and 91.8% (56/61 patients) at 48 weeks. In addition, the mean serum PSA level decreased to 3.2 *μ*g/L at 24 weeks (*n* = 60) and 5.6 *μ*g/L at 48 weeks (*n* = 86, Figure 2). Both PSA and testosterone levels were significantly less than baseline (*P* < 0.05) at 24 and 48 weeks.

Continued long-term efficacy was seen in patients who had received second, third, and fourth implants at 5 years (Figure 1). The serum testosterone level remained <30 ng/dL for 96% (24/25) of patients at two years and 100% of patients at 3 years (14/14), 4 years (9/9), and 5 years (4/4). The mean testosterone levels for years 2–5 were 11.2 ng/dL, 6.2 ng/dL, 5.9 ng/dL, and 6.5 ng/dL, respectively (Figure 1). Testosterone levels remained significantly less than baseline during years 2–5 (*P* < 0.05). Six patients had androgen-independent progression of their disease at 144 weeks (*n* = 2), 192 weeks (*n* = 3), and 240 weeks (*n* = 1). The mean serum PSA level at 2, 3, 4, and 5 years was 11.3 *μ*g/L (*n* = 51), 35.0 *μ*g/L (*n* = 22), 30.7 *μ*g/L (*n* = 13), and 132.9 *μ*g/L (*n* = 8, Figure 2), respectively. The mean serum PSA level at 2 years was not significantly different compared to baseline (*P* = 0.43); however, the mean serum PSA was significantly greater than baseline at years 3, 4, and 5 (*P* < 0.05). The median serum PSA remained <1 *μ*g/L for all 5 years.

### 3.4. Safety

Ten patients experienced difficulties with their implants during the retrospective chart review. At the time of reimplantation, the previous implant could not be located for removal in four patients. In another four patients, we clinically decided not to remove their previous implants. In one patient, the implant was reinserted after unintended expulsion less than one month after insertion. Subsequent clinical follow-up on this patient indicated maintenance of androgen suppression.

Eight patients experienced the following adverse events: redness and irritation around the implantation site (*n* = 1); pain at the implantation site 1 week after implantation, which is considered to be caused by muscle strain and is unrelated to the implant (*n* = 1); reactions to suture material (*n* = 2); and replacement of the implant due to erosion through the skin (*n* = 4). Drug related adverse effects were reported in 15% (17/113) of patients who experienced minor hot flashes, defined as tolerable/infrequent, and are consistent with patients undergoing ADT with an LHRH agonist. Twenty-six patients included in the chart review passed away after more than 5 years of evaluation; none of the deaths were related to histrelin acetate implant use.

## 4. Discussion

There is clinical interest in determining the long-term suitability of the abdomen as an insertion site for the VANTAS implant because of benefits to both the patient and the surgeon. From a surgeon's standpoint, the abdominal site is immobile, contains good anatomical landmarks, and is generally less vascular. Patients better tolerate the abdominal insertion site because it does not require strength/mobility of the shoulder joint to maintain the abducted and extended position; there is less positional risk as proximal arm insertion can cause irritation by deodorants/soaps/sweat and distal arm insertion can cause interferences from flexion/extension, and finally patients can easily visualize the abdominal site to evaluate for postprocedure wound changes. Our primary objective of this study was met because we were able to observe long-term efficacy and tolerability of the abdominal histrelin acetate subcutaneous implant used for prostate cancer management. The study's long-term data were consistent with the results from previous chart reviews regarding histrelin acetate abdominal implants and also the pivotal and extension study of the histrelin acetate arm implant [12–14].

The reviewed population in this study was similar to the population enrolled in a previous chart review, which was conducted to evaluate the abdominal implant, as well as the pivotal clinical trial and extension study, which evaluated the efficacy of histrelin acetate arm implants. In the current study, the mean patient age was 74.9 years and the percentage of Caucasian men was 90.9%. A major difference between the clinical trials of the arm implant and abdominal implant was that approximately 46% of patients in the current chart review received LHRH agonist. The clinical trials conducted by Schlegel et al. and Shore et al. to evaluate patients with arm implants excluded those who received previous ADT [13, 14].

In the current chart review, we demonstrated that the administration of the histrelin acetate implant subcutaneously into the abdomen reduced mean serum testosterone below castration levels (<50 ng/dL) for 24, 48, and 96 weeks. Data from the expanded chart review in the original abdominal implant study had mean serum testosterone values of 11.1, 16.0, and 11.2 ng/dL at 24, 48, and 96 weeks, respectively. These results are similar to the previously reported values by Siami and colleagues, who evaluated abdominal histrelin acetate subcutaneous implants. The mean serum testosterone value for 64 patients was 15.0 ng/dL at 24 weeks. In the study by Schlegel and colleagues, histrelin acetate implants were inserted for a similar duration of 52 weeks in the arm [13]. Schlegel et al. found that, in 134 patients, >99% of patients maintained chemical castration levels throughout the 52-week study duration [13].

In individuals with advanced prostate cancer, continuous long-term ADT may be clinically important. In this current chart review, we demonstrated that abdominal once-yearly histrelin acetate subcutaneous implants reduced mean serum testosterone below castration levels for 144, 192, and 240 weeks. Furthermore, the mean serum testosterone concentration remained <7 ng/dL with successive yearly cycles of insertion and removal of the abdominal implant during measurements (i.e., at 144, 192, and 240 weeks). These results are also similar to those of the study by Shore et al. In the study conducted by Shore et al., the mean serum testosterone concentration remained at <20 ng/dL for successive yearly cycles of insertion and removal for 234 weeks [14].

In our expanded chart review, a corresponding decrease in serum PSA level with testosterone level was observed in patients who had received the abdominal histrelin acetate implant during the first year. The mean serum PSA level was 3.2 and 5.6 during 24 and 48 weeks. Similar decreases were observed in both Siami et al. and Schlegel et al. [12, 13].

As expected, the natural progression of prostate cancer occurred with androgen-independent progression (AIP). AIP is defined as three consecutive increases in PSA levels after nadir. In the present study, six patients at 144 (*n* = 2), 192 (*n* = 3), and 240 weeks (*n* = 1) had AIP. Therefore, the mean serum PSA values increased above baseline to 35.0, 30.7, and 132.9 ng/dL during 144, 192, and 240 weeks, respectively. A similar progression in PSA levels was observed by Shore et al. Out of 104 patients, seven discontinued the study due to increasing PSA levels and <10% of the remaining patients experienced disease progression.

Abdominal administration of histrelin acetate was well tolerated by patients represented by the 5-year chart review and only minor adverse events were reported. Adverse events included insertion site reactions, hot flashes, and erosion of old implants through the skin. These adverse events were similar to those previously reported with the insertion of histrelin acetate implants [12–14].

The study encountered some limitations with the retrospective design. We were not able to evaluate patients' preference for the abdominal implant compared to the arm implant with a survey. Almost half of the patients (44.9%) received treatment with LHRH agonist prior to beginning the abdominal histrelin acetate yearly implant, which may confound mean testosterone levels, as patients do not recover testosterone levels immediately after stopping an agonist. Finally, the reason for patients being lost to follow-up could not be determined. Future prospective studies could help clarify some of these confounding factors in our study.

## 5. Conclusion

According to this retrospective chart review, abdominal histrelin acetate subcutaneous implants were effective on the long term at reducing serum testosterone levels below castration levels and reducing serum PSA levels. The subcutaneous abdominal insertion allows easier insertion of histrelin acetate implants for the surgeon, is well tolerated by patients, and thus is an indicated site to implant the histrelin acetate diffusion-controlled reservoir.

## Figures and Tables

**Table 1:** Summary of baseline demographics.

Demographics	Mean
Age	74.9
Testosterone (ng/dL)	106.6
PSA (*µ*g/L)	12.1

Demographics	Percent

Caucasian	90.9
African American	8.1
Hispanic	1.0
Testosterone ≤ 30 ng/dL	48.5
PSA ≥ 5 *µ*g/L	55.7
Previous LHRH agonist	44.9
